# Clinical characteristic, red blood cell indices, iron profile and prognosis of heart failure in females

**DOI:** 10.21542/gcsp.2021.13

**Published:** 2021-06-30

**Authors:** Surender Deora, Jai Bharat Sharma, Shubham Kumar Sharma, Nikhil Chaudhary, Atul Kaushik, Rahul Choudhary, Jaykaran Charan, Deepak Kumar, Gopal Krishna Bohra, Kuldeep Singh

**Affiliations:** 1 Department of Cardiology, All India Institute of Medical Sciences, Jodhpur, Raj, India; 2 Department of Pharmacology, All India Institute of Medical Sciences, Jodhpur, Raj, India; 3 Department of General Medicine, All India Institute of Medical Sciences, Jodhpur, Raj, India; 4 Professor & Dean Academics, All India Institute of Medical Sciences, Jodhpur, Raj, India

## Abstract

**Background:** Heart failure is a leading killer worldwide, with concurrent anaemia and iron deficiency portending sepulchral prognosis. Anaemia is rampant, with 53% prevalence in Indian females, but iron deficiency can be present even without anaemia. Therefore, this study was planned to determine the clinical profile, red blood cell indices, and effects of iron deficiency, on the course and prognosis of heart failure in Indian females.

**Materials and methods:** This was a hospital-based observational study, conducted at a tertiary care teaching institute in India. Data from 147 females enrolled in the study between September 2017 to March 2020 was collected out of all patients enrolled in ongoing heart failure registry at the institute. Clinical characteristics at presentation, iron profile, red blood cell indices, treatment and mortality data was collected.

**Results:** Mean age of the subjects (*n* = 147) was 53.31 ± 17.1 years with 55% non-rheumatic and 45% with rheumatic heart disease. The patients with rheumatic heart disease were younger, with a higher prevalence of atrial fibrillation. Non-rheumatic patients had a higher prevalence of CV risk factors like diabetes, hypertension, renal failure, more patients in NYHA IV, and 83% patients had LVEF ≤40%. Anaemia was present in 49%, however iron deficiency was present in 89% (absolute iron deficiency in 80% and functional iron deficiency in 9%) with no significant difference between rheumatic and non-rheumatic group. Red blood cell indices showed no significant difference across the spectrum of iron deficiency and anaemia, except lower mean corpuscular volume in patients with both iron deficiency and anaemia. The mean survival time was 840 days, with no significant difference between groups. There was significantly higher mortality in patients with iron deficiency (log rank 0.045).

**Conclusion:** Iron deficiency–with or without anaemia–is very high in Indian females, worsening survival in heart failure. Proper diagnosis with iron supplementation will improve the prognosis.

## Introduction

Heart failure (HF) is a leading cause of morbidity and mortality worldwide and is a major burden on public health system. It is the primary cause of hospitalisation in patients above 65 years of age with re-admission rates of 38% in the first month and 43% in 6–12 months^1^. The post-admission mortality of HF varies between 20–30%. In India, with limited and sparse data, the prevalence of HF is estimated to vary from 1.3 to 23 million^2^. With the increase in the incidence and prevalence of diabetes, hypertension and dyslipidemia in India, there is increased trend of patients with non-rheumatic HF as noted in the Trivandrum Heart Failure registry^2^. The INDUS study of HF in rural India revealed the prevalence of 1.2/1000 of population. In this study, diastolic HF was main cause of HF with rheumatic heart disease (RHD) as the most common etiology followed by coronary artery disease (CAD)^3^.

Although HF affects both genders equally, females at an older age are at greater risk than males^4^. Above 65 years, the incidence rate of HF triples for females, whereas it only doubles for males^5^. The type of HF varies with gender, with heart failure with reduced ejection fraction (ejection fraction < 40%; HFrEF) more common in males, but heart failure with preserved ejection fraction (ejection fraction > 50%; HFpEF) is twice as common in females^6^.

Anaemia is another major public health challenge in India, along with HF, especially in females and has huge impact on the course and prognosis of HF patients. According to National Family Health Survey 2015–2016, the prevalence of anaemia in Indian females is as high as 53%^7^. Anaemia and iron deficiency (ID) are associated with poor outcomes in HF patients and with such a high prevalence of anaemia in Indian females, the course and prognosis of these patients are different from HF in males.

In developing countries like India, the increasing rates of HF will put excess burden on an already stretched health care system fighting with the diseases like rheumatic heart disease, untreated congenital heart disease and other infectious diseases. Previous studies, with males as the major group, have shown prevalence of iron deficiency of 50–70%. To better utilise the available resources for managing the patients of HF, real-world data on clinical characteristics, etiology, and course of HF, with prognosis, is needed–and this is lacking for HF in Indian females. Therefore, the study was planned to determine the clinical profile, red blood cell indices, iron deficiency, and their effect on the course and prognosis of HF in Indian females.

## Methods

This was hospital-based observational study conducted at a tertiary care teaching institute in India. Data from 147 females enrolled in the study between September 2017 to March 2020 were collected out of all patients enrolled in ongoing heart failure registry at the institute. This included both Out Patient Department (OPD) and In-Patient Department (IPD) patients from the departments of Cardiology, Trauma and Emergency, and Internal Medicine. All females older or equal to 18 years of age, presenting with sign and symptoms of HF with New York Heart Association (NYHA) class II–IV, were included for analysis. HF was diagnosed based on the presence of symptoms and signs of HF and according to latest guideline by American Heart Association/American College of Cardiology/European Society of Cardiology^8,9^.

Demographic details, clinical characteristics, red blood cell indices, and iron studies were collected from the registry details. All patients were evaluated by electrocardiogram (ECG), echocardiography, and other laboratory investigations such as renal function test and liver function test. Patients were divided as per the etiology of HF as rheumatic and non-rheumatic. Left Ventricular Ejection Fraction (LVEF) was measured using Teichholz formula and were assigned to either heart failure with ejection fraction < 40% (HFrEF), or heart failure with ejection fraction ≥ 40%. Hypertension was defined as blood pressure more than 140/90 mm Hg and diabetes was defined as fasting blood sugar ≥ 126 mg/dL or HBA1c ≥ 6.5%.

Anaemia was defined according to WHO definition of haemoglobin (Hb) concentration of < 12 mg/dL in females. Absolute iron deficiency (ID) was defined as serum ferritin < 100 μg/dL, whereas functional iron deficiency was defined as serum ferritin 100–299 μg/dL and transferrin saturation (TSAT)< 20%. TSAT was calculated as a ratio of serum iron and total iron binding capacity (TIBC), multiplied by 100. Red cell distribution width (RDW), a parameter of anisocytosis is measured both in absolute value as the standard deviation (SD) of erythrocyte volumes (RDW-SD), or as the coefficient of variation (RDW-CV) [i.e., (Standard deviation of MCV ÷ MCV) × 100]. The normal range of RDW-CV is 11.5–14.5% and of RDW-SD is 40–55 fL.

Heart failure patients with acute coronary syndrome, other comorbidities like advanced chronic kidney disease, chronic lung disease, chronic liver disease, malignancy, blood or iron transfusion in the previous 3 months, taking drugs for anaemia, and pregnancy, were excluded from the study.

Patients were followed in physical OPD or by telemedicine and all clinical details were recorded with their functional status according to NYHA functional classification, number of re-hospitalisations, and mortality–if any. The study was approved by the Institutional Ethics Committee and all patients gave written informed consent.

### Statistical analysis

The statistical analysis was performed using SPSS version 25 (IBM, Armonk, New York). Baseline clinical characteristics, haemoglobin, iron, and red cell indices were analysed and compared according to the etiology of HF. Categorical variables were expressed as a number and a proportion (%) and the inter group difference was tested using chi-square test. Continuous variables were tested for normal distribution and expressed as mean ±  standard deviations for data with normal distribution and median (95% confidence interval) for skewed data. Difference between the groups was analysed using Student t test for comparison of means and Mann-Whitney U test for comparison of data without normal distribution. Kaplan-Meier survival analysis was used to evaluate impact of etiology of HF and ID state on survival. Two tailed *P*-value of < 0.05 was considered statistically significant.

## Results

A total of 147 females with HF were included in the study, out of which 66 had rheumatic and 81 had non-rheumatic etiology (ischemic cardiomyopathy, idiopathic and post-partum cardiomyopathy) (Table 1). There was a significant difference in mean age of the patients between the two groups (42 years, rheumatic and 62 years, non-rheumatic; *p* < 0.001). There was also significant difference in cardiovascular risk factors like diabetes (*p* = 0.0003), hypertension (*p* = 0.0019) and renal failure (*p* = 0.03), all were more common in heart failure with non-rheumatic etiology. Regarding the presence of atrial fibrillation, it was more commonly observed in patients with rheumatic etiology (31%) as compared to patients with non-rheumatic etiology (12%) with highly significant statistical difference (*p* < 0.0001). The functional class NYHA IV and HFrEF (LVEF < 40%) was observed more commonly in patients with non-rheumatic HF, with significant statistical difference when compared with rheumatic HF (NYHA IV, *p* = 0.0029; LVEF< 40%, *p* < 0.0001). The compliance to medications was poor with 44% of all females with HF were on angiotensin-converting enzyme inhibitors (ACE-I) and 62% were on beta blockers (BBs). The use of ACE-I was more common in patients with non-rheumatic HF(59%), whereas beta blockers were more commonly used in patients with rheumatic HF (88%).

**Table 1:** Clinical characteristic and anaemia profile of patients according to etiology of heart failure.

	All patients (*n* = 147)	Rheumatic (*n* = 66)	Non-Rheumatic (*n* = 81)	*P* value
Age, in years (mean (SD))	53.31 (17.1)	42 (14)	62 (14)	<0.0001
Renal failure % (n)	11 (16)	5 (3)	16 (13)	0.03
Diabetes % (n)	10 (15)	0	18 (15)	0.0003
Hypertension % (n)	12 (18)	3 (2)	20 (16)	0.0019
NYHA Class % (n)				
I	15 (22)	15 (10)	15 (12)	1
II	61 (90)	67 (44)	57 (46)	0.22
III	15 (22)	17 (11)	13 (11)	0.49
IV	9 (13)	1 (1)	15 (12)	0.0029
Atrial fibrillation % (n)	29 (43)	47 (31)	15 (12)	<0.0001
ACE inhibitors % (n)	44 (65)	26 (17)	59 (48)	0.0001
Beta blockers % (n)	62 (91)	88 (58)	41 (33)	<0.0001
LVEF < 40%, % (n)	48 (70)	5 (3)	83 (67)	<0.0001
Anaemia % (n)	49 (72)	48 (32)	49 (40)	0.90
Absolute ID % (n)	80 (117)	82 (54)	78 (63)	0.55
Functional ID % (n)	9 (13)	11 (7)	7 (6)	0.39

Anaemia was seen in 49% of all patients, but cumulative ID was detected in 89% of all patients (absolute ID in 80% and functional ID in 9%), with statistically no difference according to etiology of HF. When detailed analysis of haemoglobin, iron, ferritin, and RBC indices was done according to different haematological groups, it was observed that out of all 147 patients, 7.5% had no anaemia and no ID, whereas anaemia without ID, ID without anaemia, and both anaemia and ID, were seen in 4%, 43.5% and 45% respectively (Table 2). Interestingly, 43.5% of patients had significantly lower iron and ferritin levels, but normal haemoglobin, when compared with patients without anaemia and ID.

**Table 2:** Haemoglobin, iron and red cell indices according to presence of anaemia and/or iron deficiency.

	All patients (*n* = 147)	No ID and No Anaemia (*n* = 11)	Anaemia only (*n* = 6)	ID only (*n* = 64)	ID and Anaemia (*n* = 66)
Haemoglobin, g/dL (SD)	12.1 (11.7–12.5)	13.4 (12.3–14.8)	10.6 (9.2–11.2)^***^	12.9 (12.8–13.4)	11 (10.8–11.4)^***^
HCT, %	38.63 (5.06)	43.6 (3.8)	33.5 (3.9)^***^	42.1 (2.4)	34.9 (4.0)^***^
Iron, μg/dL	50 (44–58)	75 (58–90)	60 (43–71)	60 (51–70)*	31.5 (28–42)^***^
Ferritin, μg/dL	36.3 (26.4–51)	306 (143–427)	134 (131–477)	46 (29–57)^***^	21 (17–25)^***^
TIBC, μg/dL	364 (348–388)	339 (294–368)	300 (273–322)	363 (335–391)	390 (362–418)^*^
TSAT %	14.6 (12.1–16.6)	21.4 (20.3–25.9)	21.8 (20.2–23.8)	16.3 (14.6–18.8)^**^	8.6 (6.4–10.7)^***^
MCV, fL	85.05 (7.6)	88.3 (4.5)	84.7 (12.9)	87 (5.5)	82.7 (8.6)^*^
MCH, pg	26.9 (26.5–17.4)	26.5 (26.2–28.2)	28.2 (27.3–29.1)	27.6 (27.1–28)	26.1 (25.1–26.6)
MCHC, g/dL	31.2 (30.9–31.4)	31 (29.9–31.8)	31.2 (30.7–32)	31.5 (31.4–31.7)	30.4 (30.1–31.3)
RDW-CV %	14.5 (14.3–15.1)	14.5 (12.3–16.5)	13.9 (12.3–15)	13.6 (13.2–14.3)	15.7 (14.6–16.4)
RDW-SD, fL	44.8 (43.8–46.3)	47.1 (42–52)	43.2 (39.8–49.2)	43.7 (42.9–45.1)	47.0 (44.5–49.2)

**Notes.**

*P* value for comparison with the group ‘No ID and no anaemia’.

* *p* < 0.05.

** *p* < 0.01.

*** *p* < 0.001.

**Figure 1.**
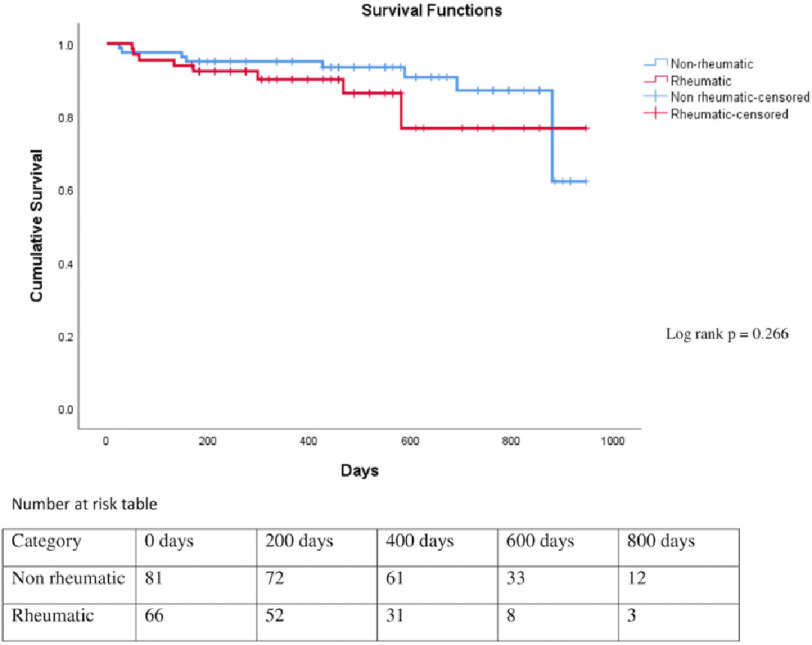
Kaplan Meier survival curve of patients with heart failure with rheumatic and non-rheumatic etiology (log rank *p* = 0.266).

**Figure 2.**
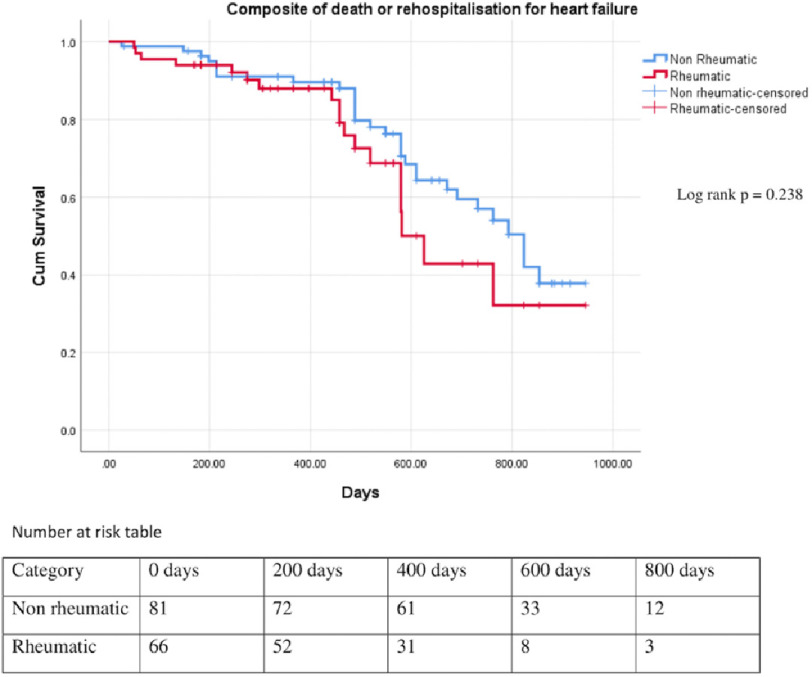
Kaplan Meier survival curve of patients with heart failure with rheumatic and non-rheumatic etiology for composite of death or rehospitalisation (log rank *p* = 0.238).

**Figure 3.**
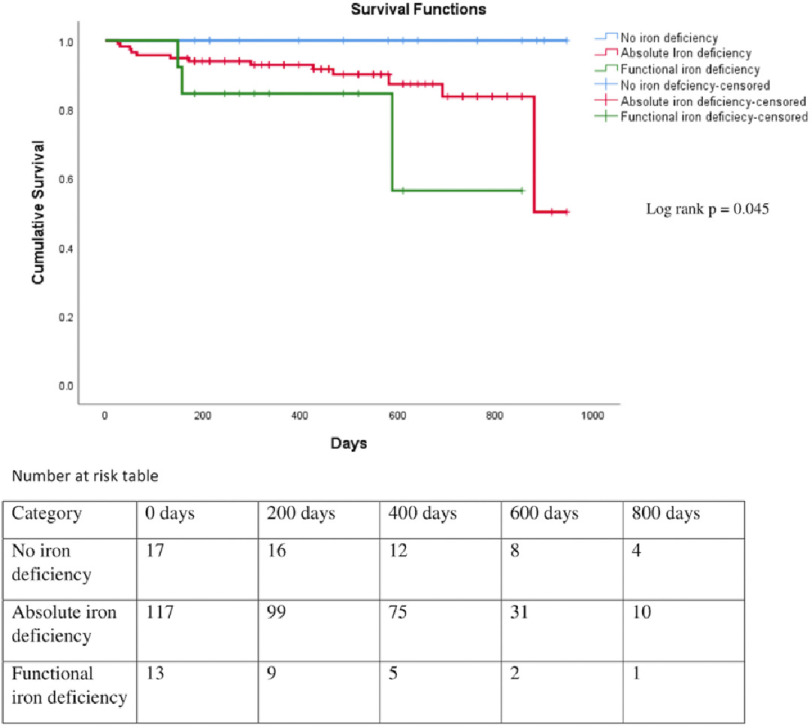
Kaplan Meier survival curve of patients with heart failure with absolute or functional iron deficiency and without iron deficiency (lfigog rank *p* = 0.045).

Total median follow-up was of 488 days (rheumatic, 381 days and non-rheumatic, 564 days). Of patients with rheumatic HF, 27% had rehospitalisation and 12% had mortality, whereas patients with non-rheumatic HF, 37% had rehospitalisation and 11% mortality during the follow up. There was no statistically significant difference in rehospitalisation, mortality, and in composite of both, among patients with rheumatic and non-rheumatic etiology (Table 3; Figures 1 and 2). There was significant difference in the rate of mortality in patients with absolute or functional ID when compared with patients without anaemia and ID (*P* = 0.045) (Figure 3).

**Table 3:** Follow up and mortality of patients according to etiology of heart failure.

	Total	Rheumatic (*n* = 66)	Non rheumatic (*n* = 81)	*P* value
Duration of heart failure (median, in days (95% CL))	671 (610–732)	610 (549–732)	732 (732–976)	0.782
Follow up (Median, in days (95% CL))	488 (457–564)	381 (298–457)	564 (488–610)	0.000
Rehospitalisation, % (n)	33 (48)	27 (18)	37 (30)	0.1995
Mean survival time (days)	840	813	859	0.266
Mortality, % (n)	12 (17)	12 (8)	11 (9)	0.85

## Discussion

The present study was performed to determine the clinical characteristics, red blood cell indices, and prognosis of HF in Indian females, and also the impact of anaemia–with or without ID–on prognosis. The clinical characteristic and etiology of HF are important as RHD is still prevalent in India and the appropriate management and intervention given early in the course of the disease will improve the chances of survival. There is a dearth of data from India with regards to epidemiology, clinical characteristics, and prognosis of heart failure. This study is first of its kind in India where only females with HF were assessed. The Trivandrum Heart Failure Registry (THFR) recruited 1232 patients out of which 371 were females. Out of 371 females in their registry, there was significant difference in LV systolic function with 33% of patients had LVEF ≥ 45% and 67% had LVEF < 45%^10^.

In our study, LVEF < 40% was chosen as criteria to diagnose HFrEF and was found in 48% of females with LVEF < 40%. Most of our patients were symptomatically stable with only 9% in NYHA class IV as compared to 34.5% in THFR. The most common etiology of HF in females in THFR was ischemic heart disease (IHD), seen in 66% of patients, whereas RHD constituted only 11.6%, as compared to 45% of females with RHD in our study^11^.

However, the trend toward increases in non-rheumatic etiology of HF in females was observed in our study, which constituted 55% of all patients. This included HFrEF-like ischemic cardiomyopathy, idiopathic dilated cardiomyopathy, peripartum cardiomyopathy, and HFpEF-like congenital heart disease, diabetic or hypertensive cardiomyopathy. Comorbidities like diabetes, hypertension, renal failure was seen in 59%, 53% and 15% of patients in THFR, whereas these comorbidities were seen in 10%, 12% and 11% of our patients, which again may be because of relatively more patients with RHD.

Atrial fibrillation was also more common in our study. It was seen in 29% of patients, compared to THFR (18.3%) which may be due to a greater number of RHD patients in our study. The compliance to medications was poor in THFR, with only 45% on ACE-I and 54% on BBs in overall patients with HF without gender bias, whereas in our study exclusively on females, the use was 44% and 62%, respectively. In both studies, the low use of guideline directed medical treatment (GDMT) is a major concern, as both of the drugs decrease mortality in patients with HFrEF.

Gender and ethnicity are an independent correlate of ID in patients with HF. In a study by Yeo et al in 751 patients of HF, ID was more prevalent in females as compared to males (70.5% vs. 58.6%, *P* = 0.004)^12^. In the same study, Indians females with HF have been found to have higher prevalence of ID even after correcting for clinical and statistical characteristics as compared to their Chinese and Malay counterpart (81.6 vs. 62.9 vs. 58.1% in HF, *P* = 0.001). In our study, the overall prevalence of anaemia was 49% in females, whereas ID was seen in 89% of patients. Notably, 43.5% had only ID without anaemia. With the mean age of the patients 53 ±  17 years, menstrual loss can’t fully explain the high prevalence of ID in these females. Other nutritional factors e.g., high consumption of tea, vegetarianism, and less access/affordability of iron rich food may be contributing.

There are no studies of red cell indices and iron deficiency exclusively in Indian females with HF. The study by Arora et al recruited 275 patients for studying anaemia profile in HF, out of which 31.6% were females^13^. Overall, ID was diagnosed in 53.8% of patients with HF and it was seen in 61.6% of patients with anaemia, and 28.1% of patients without anaemia. Similarly, the study by Sharma et al estimated the prevalence and pattern of ID in 150 patients of HF with or without anaemia^14^. In their study, only 32% were females but ID was seen in 91.6% of these females.

The effect of ID on long-term mortality was studied by Tkaczyszyn et al in 1,821 patients with HF, with 29% female^15^. In this study, the authors concluded that ID had a detrimental impact on long-term survival, which was independent of red cell indices. Similar to their study, we also found adverse impact of ID on the prognosis of the females with HF. As ID is more common in Indian females, adequate dietary supplements and treatment of ID may change the prognosis of HF.

## Limitations

There are several limitations of our study. First, a major limitation was that it was a single-centre observational study. India is a large country, with varied geographical, cultural and dietary differences, therefore the prevalence of anaemia and/or iron deficiency may differ across the country. Large, multicentre studies from various geographical locations will help in better understanding this phenomenon.

A second limitation is the small number of patients in the study, which may underestimate the effect of other red cell indices on the prognosis of HF patients.

Thirdly, most of the follow-up of these patients was telephone-based because of the ongoing COVID-19 pandemic, therefore the exact cause of death is not known in these patients.

## Conclusions

Our study is the first of its kind from India including only females with HF. The study suggests that with an increase in prevalence of cardiometabolic risk factors, like diabetes, hypertension etc., non-rheumatic cause of HF has increased. There is poor compliance to medications, which is impacting and aggravating the poor prognosis in these patients and therefore needs involvement of a HF counsellor, along with nursing care and medical consultation. The ID with or without anaemia is very high in Indian females, and proper diagnosis with iron supplementation will improve the prognosis.
